# Assessment of monosodium glutamate (MSG) intake in a rural Thai community: questioning the methodological approach

**DOI:** 10.1186/1743-7075-10-52

**Published:** 2013-07-26

**Authors:** Karuthan Chinna, Tilakavati Karupaiah

**Affiliations:** 1 Julius Centre, University of Malaya, Kuala Lumpur 50606, Malaysia; 2 Dietetics Program, Faculty of Health Sciences, National University of Malaysia, Jalan Raja Muda Abdul Aziz, Kuala Lumpur 50300, Malaysia

**Keywords:** Monosodium glutamate, Dietary assessment, Metabolic syndrome, Overweight

## Abstract

We examined the methodological approach to the assessment of monosodium glutamate intake. The high carbohydrate and low fat consumption characteristic of this study population would be conducive to the development of metabolic syndrome. However, anomalies in the assessment of dietary information limits conclusion to a causal link of monosodium glutamate to metabolic syndrome and overweight because the study lacks data on the main dietary patterns of consumption. Given the current paucity of data from human studies on monosodium glutamate intake and risk, more studies with robust methodology are required to assess causal links to disease.

## Letters to the editor

Dear Editor,

The paper by Insawang *et al*.*,* concerning monosodium glutamate (MSG) intake and its association with metabolic syndrome (Met-S) in a rural Thai population is the centre of a current debate [1-3]. They estimated for every 1 g increase in MSG consumption, Met-S risk increased with an odds ratio (OR) of 1.14 (CI 1.12-1.28) or being overweight with an OR of 1.16 (CI 1.04-1.29).

### Was the novel approach in assessment of MSG justified?

The ability of cognitive recall to capture accurately micro-quantities of substances in the food chain is subject to interpretation error. The InterMap Study reported a mean intake of 0.33 g/day by participants demonstrating the amount of MSG used in food preparation which was then weighed [4]. In the China Health and Nutrition Survey the MSG container was weighed before cooking started and at the end of the day, yielding an estimate of 1.8 g/day [5]. MSG consumption of 3.8 g/day in the Jiangsu Nutrition Study (JNS) was estimated from total monthly consumption reported per household divided by the number of residents and adjusted by proportion of household energy intake for each individual [6]. In these 3 studies, assessment for total glutamate content included both direct and indirect sources [4-6].

Insawang et al. provided each household a 250 g box of MSG and difference in box weight from the last (10^th^ day) to the 1^st^ day was assumed to be the quantity of MSG consumed after factoring in household number averaged over the number of days (g/person/day) [1]. This method would be an adaptation of the food disappearance approach [7]. This term, as defined by the USDA-ERS, means difference between beginning inventories and ending food stocks [8]. Using supermarket shopping receipts to assess food supply coming into a household or even the method of monitoring stocks by storekeepers are similar adaptations [9,10]. The possibility of random error would exist because of the exclusion of subjects ≤ 10 years from the count (overestimation), if leftovers and food are given away (overestimation), limited number of assessment days, and lack of accounting for ‘hidden’ sources of MSG (underestimation). A further possibility is systematic error rising from subject bias as the MSG product was given free raising a core question- *were the participants using ‘higher than normal’ amounts of MSG in cooking?* However, to satisfactorily establish a causal diet-disease relation, evidence must be examined from a variety of sources and congruence between these sources achieved [11].

### Was the research question adequately addressed?

Two anomalies in the reported nutrient intake data are present:

i. About 66.2% of subjects were engaging in vigorously active lifestyle with a median consumption of 2032 kcal comprising ~66% carbohydrate and ~14.7% fat calories and this pattern was consistent at the highest tertile of MSG intake (68% carbohydrate and 14.1% fat calories). Direct conversion of macronutrients to calories at the highest tertile indicates carbohydrate consumption reaching ~71% calories. The high carbohydrate and low fat consumption characteristic for this population typifies Pattern 4 diet of nutrition transition taking place in developing countries, which despite a high-activity profile and lean body phenotype would be conducive to the development of Met-S [12]. These dietary factors with causal links to MET-S were not adjusted as confounding factors in the final analysis [1]. The JNS study demonstrated, after adjusting for either rice intake or dietary patterns, no association between MSG intake and weight gain could be found [6].

ii. Systematic error is probable in the dietary assessment. The number of subjects with BMI >25 kg/m^2^ was increasing significantly across tertiles (p=0.021) but yet caloric intake did not reflect this trend suggesting underreporting [1]. MSG use is related to its pleasurable taste sensation [13]. Obese women have been shown to have lower taste sensitivity for MSG and therefore prefer higher concentrations than normal weight women [14].

### Was statistical interpretation correct?

The presentation of statistics data for OR and confidence intervals (CI) for study outputs as reported by the Insawang et al. in Table two is misleading [1]. The title of their table cited OR for MSG intake with insulin resistance, overweight and Met-S as predictors. Yet overweight and Met-S were identified as significant predictors of MSG. This is totally wrong. MSG depends on Met-S or is it *vice versa*?

*What is the significance of OR*? The problem was the way the statistical information was presented by Insawang et al. [1]. It is true that if the exposure (MSG intake) and the outcome (having metabolic syndrome) are both dichotomous, an OR value of 1.14 (95% CI 1.12 - 1.28) is very close to one and of hardly any clinical relevance. It is also true that with a big sample size even a small OR will be significant. However, if the predictor is a continuous variable and the outcome is categorical, an OR value of more than zero indicates an increased risk. If this is the case, then we accept that the lower limit of 1.14 is an indication of increasing risk. This argument will be the same for the outcome of being overweight (odds ratio 1.16, 95% CI 1.04 - 1.29).

## Conclusions

Methodological limitations in the study relate to the assessment of MSG and dietary macronutrients as well as the interpretation of the clinical relevance of MSG exposure to outcomes. The direction of animal studies link MSG-linked obesity to Met-S, diabetes and liver disease [15,16]. Therefore more human studies are needed to explain a cause-effect relationship and mechanisms of action as to whether MSG causes weight gain and insulin resistance separate from the larger macronutrient matrix and lifestyle factors. Given these loose threads of evidence the spirit of scientific enquiry should persist, as evidenced from the developmental pathways of animal to human studies establishing *tran*s fatty acids as a risk factor for cardiovascular disease [17,18].

## Abbreviations

MSG: Monosodium glutamate; MET-S: Metabolic syndrome; OR: Odds ratio; CI: Confidence interval; JNS: Jiangsu nutrition study.

## Competing interests

The authors declare that they have no competing interests.

## Authors’ contributions

KC and TK contributed intellectual insights into this critique. Both discussed, debated and drafted this critique jointly. Both authors read and approved the final manuscript.

## References

[B1] Insawang T Selmi C Cha’on U Pethlert S Yongvanit P Areejitranusorn P Boonsiri P Khampitak T Tangrassameeprasert R Pinitsoontorn C Prasongwattana V Gershwin ME Hammock BD Monosodium glutamate (MSG) intake is associated with the prevalence of metabolic syndrome in a rural Thai population Nutr Metab 2012 9 50 10.1186/1743-7075-9-50 PMC3583269 22681873

[B2] Collison KS Commentary on “Further studies are necessary in order to conclude a causal association between the consumption of monosodium L-glutamate (MSG) and the prevalence of the metabolic syndrome in the rural Thai population” Nutr Metab 2013 10 13 10.1186/1743-7075-10-13 PMC3599074 23339656

[B3] Rogers MD Further studies are necessary in order to conclude a causal association between the consumption of monosodium L-glutamate (MSG) and the prevalence of metabolic syndrome in the rural Thai population Nutr Metab 2013 10 14 10.1186/1743-7075-10-14 PMC3599553 23347668

[B4] He K Zhao L Daviglus ML Dyer AR Van Horn L Garside D Zhu L Guo D Wu Y Zhou B Stamler J and for the INTERMAP Cooperative Research Group Association of monosodium glutamate intake with overweight in Chinese adults: the INTERMAP Study Obesity (Silver Spring) 2008 16 1875 1880 10.1038/oby.2008.274 18497735 PMC2610632

[B5] He K Du S Xun P Sharma S Wang H Zhai F Popkin B Consumption of monosodium glutamate in relation to incidence of overweight in Chinese adults: China Health and Nutrition Survey (CHNS) Am J Clin Nutr 2011 93 1328 1336 10.3945/ajcn.110.008870 21471280 PMC3095503

[B6] Shia Z Luscombe-Marsha ND Witterta GA Yuana B Daia Y Pana X Taylor AW Monosodium glutamate is not associated with obesity or a greater prevalence of weight gain over 5 years: findings from the Jiangsu nutrition study of Chinese adults Brit J Nutr 2010 104 457 463 10.1017/S0007114510000760 20370941

[B7] Nelson M Bingham SA Margetts BM, Nelson M Assessment of food consumption and nutrient intake Design Concepts in Nutritional Epidemiology 2003 2 Oxford: Oxford University Press 123 169

[B8] USDA, Economic Research Service Frazao E America’s eating habits: changes and consequences 1997 Washington, D.C: Agricultural Information Bulletin No. 750 http://www.fda.gov/Food/GuidanceRegulation/GuidanceDocumentsRegulatoryInformation/IngredientsAdditivesGRASPackaging/ucm074725.htm#fooding

[B9] Ransley JK Donnelly JK Botha TN Greenwood DC Cade JE Use of supermarket receipts to estimate energy and fat content of foods purchased by lean and overweight families Appetite 2001 41 141 148 14550311 10.1016/s0195-6663(03)00051-5

[B10] Payne-Palacio J Theis M Foodservice Management: Principles and Practices 2011 12 United States: Prentice Higher Education (US) 600

[B11] Tarasuk VS Brooker A Interpreting epidemiologic studies of diet disease relationships J Nutr 1997 127 1847 1852 9278571 10.1093/jn/127.9.1847

[B12] Misra A Khurana L Obesity and the metabolic syndrome in developing countries J Clin Endocrinol Metab 2008 93 11 9 30 10.1210/jc.2008-1595 18987276

[B13] Bellisle F Glutamate and the UMAMI taste: sensory, metabolic, nutritional and behavioural considerations. a review of the literature published in the last 10 years Neuroscience & Biobehavioral Rev 1999 23 23 438 9989429 10.1016/s0149-7634(98)00043-8

[B14] Yanina Pepino Y Finkbeiner S Beauchamp GK Mennella JA Obese women have lower monosodium glutamate taste sensitivity and prefer higher concentrations than do normal weight women Obesity (Silver Spring) 2010 18 959 965 10.1038/oby.2009.493 20075854 PMC2987588

[B15] Nakanishia Y Tsuneyamaa K Fujimotoc M Salungaa TL Kazuhiro K Ana J-L Takanoa Y Iizukae S Nagatae M Suzukie W Shimadae T Aburadae M Nakanof M Selmig C Eric Gershwin NE Monosodium glutamate (MSG): a villain and promoter of liver inflammation and dysplasia J Autoimmunity 2008 30 42 50 10.1016/j.jaut.2007.11.016 18178378

[B16] Chen W Wang L-L Liu H-Y Long L Li S Peroxisome proliferator-activated receptor δ-agonist, GW501516, ameliorates insulin resistance, improves dyslipidaemia in monosodium l-glutamate metabolic syndrome mice Basic & Clin Pharmaco & Toxic 2008 103 240 246 10.1111/j.1742-7843.2008.00268.x 18684227

[B17] Willett WC Ascherio A Trans fatty acids: are the effects only marginal? Am J Public Health 1994 84 722 724 10.2105/AJPH.84.5.722 8179036 PMC1615057

[B18] Mozaffarian D Katan MB Ascherio A Stampfer MJ Willet WC *Trans* fatty acids and cardiovascular disease N Engl J Med 2006 354 1601 1613 10.1056/NEJMra054035 16611951

